# A Randomized Pilot Study of Acceptance and Commitment Therapy to Improve Social Support for Veterans with PTSD

**DOI:** 10.3390/jcm11123482

**Published:** 2022-06-17

**Authors:** Megan M. Kelly, Erin D. Reilly, Victoria Ameral, Stephanie Richter, Seiya Fukuda

**Affiliations:** 1 VISN 1 New England Mental Illness Research, Education, and Clinical Center, VA Bedford Healthcare System, Bedford, MA 01730, USA; erin.reilly@va.gov (E.D.R.); victoria.ameral@va.gov (V.A.); stephanie.richter@va.gov (S.R.); seiya.fukuda@va.gov (S.F.); 2 Department of Psychiatry, University of Massachusetts Chan Medical School, Worcester, MA 01655, USA

**Keywords:** PTSD, social impairment, social functioning, social support, relationships, acceptance, mindfulness

## Abstract

Veterans with PTSD often have substantial interpersonal difficulties and low levels of social support, which puts them at increased risk of mortality, but few treatments address global social impairment for veterans with PTSD. This study is a pilot randomized trial of Acceptance and Commitment Therapy to Improve Social Support for Veterans with PTSD (ACT-SS), a psychotherapy that targets social avoidance and eroded social relationships, compared to Person-Centered Therapy (PCT), a non-directive psychotherapy. Participants were randomized to twelve sessions of either ACT-SS (*n* = 21) or PCT (*n* = 19). The results showed that veterans with PTSD had high ratings of satisfaction for both treatments. Contrary to the PCT group, participants in the ACT-SS group showed a significant improvement in the quality of social relationships, engagement in social and leisure activities, and PTSD symptoms from the baseline assessment to the end of treatment and a three-month follow-up. Veterans in the ACT-SS group, but not the PCT group, also showed significant improvements in mindfulness and valued living and a reduction in experiential avoidance from baseline to the end of treatment, with sustained improvements in valued living at the three-month follow-up. Overall, the present study demonstrated the feasibility, acceptability, and positive preliminary outcomes of ACT-SS for veterans with PTSD.

## 1. Introduction

Low social support is a key factor related to serious negative health outcomes, including poor physical and mental health, increased mortality risk, and suicidal ideation and behavior [1,2,3,4]. Social relationships buffer people from stress, improve their mental health [1,5,6], and provide meaningful roles that increase self-esteem and purpose in life [7,8]. Veterans with PTSD often have substantial interpersonal problems and low levels of social support from family, partners, and peers, resulting in problems with social reintegration [9]. For example, Operation Iraqi Freedom/Operation Enduring Freedom/Operation New Dawn (OIF/OEF/OND) veterans, for whom prevalence estimates of PTSD range from 17% to 21% [10,11], reported a fourfold increase in rates of interpersonal conflict within six months of returning from deployment [12]. Critically, social support is also linked with suicidal ideation and behavior. Veterans with PTSD have rates of suicide that are five times higher than the general population [13], making reducing social isolation among these veterans imperative. Interventions that specifically target improving social support for veterans with PTSD are critically needed to improve these veterans’ health, well-being, and survival [1].

The negative outcomes associated with poor social support are of particular concern for veterans with PTSD, who often perceive the world to be dangerous, view their social support network as a threat to their safety, and avoid members of their support network in order to increase their perceived safety [14,15]. Avoidance of social contact among people experiencing PTSD symptoms may be conceptualized as experiential avoidance within psychological flexibility theory [16,17]. Psychological flexibility theory maintains that experiential avoidance interferes with living a life consistent with one’s values [18], which, for many people, includes maintaining relationships with family, partners, friends, peers, and others [17]. Veterans with PTSD who perceive others as threatening may withdraw to avoid the anxiety they experience when interacting with others [19]. Veterans with PTSD frequently act aggressively to control or avoid interactions with others (i.e., another form of experiential avoidance) [19,20], leading to further decline in support over time. Experiential avoidance has been shown to be associated with both greater PTSD symptom severity and lower perceived social support in Iraq and Afghanistan veterans [21]. Further, social avoidance has been shown to mediate the association between PTSD and partner satisfaction in Iraq and Afghanistan war veterans [22]. In a study of 145 veterans who served during the conflicts in Iraq and Afghanistan, experiential avoidance was found to be a significant mediator between PTSD symptoms and social support in veterans [23]. There is also evidence that social support (and, conversely, social problems) influences outcomes from PTSD treatment approaches, such as cognitive processing therapy [24,25]. New treatment approaches with a specific focus on improving the quality of social relationships for veterans with PTSD are needed to help improve their quality of life.

Acceptance and Commitment Therapy (ACT) may be a useful therapeutic approach for veterans with PTSD and social relationship difficulties. ACT is an evidence-based treatment that focuses on identifying a person’s valued life goals and explicitly targets experiential avoidance to assist clients in committing to behavior change aligned with their values and becoming more psychologically flexible [26,27,28,29,30]. There is strong empirical evidence to show that ACT improves several conditions, with robust evidence for the treatment of pain, depression, anxiety disorders, and psychosis [31,32,33]. ACT has also shown preliminary evidence in improving interpersonal difficulties [34,35,36] and PTSD symptoms [37,38,39,40]. However, ACT has not yet been specifically evaluated in its effects on social relationship difficulties for veterans with PTSD.

The present paper presents the results of a small randomized clinical trial of a targeted ACT intervention for veterans with PTSD compared to Person-Centered Therapy, a non-directive treatment. The primary aim was to evaluate feasibility and acceptability of Acceptance and Commitment Therapy to Improve Social Support for Veterans with PTSD (ACT-SS) [41]. Secondary aims included preliminary examinations of treatment outcomes, including quality of life, quality of social relationships, social support, engagement in social and leisure activities, PTSD symptoms and evaluations of potential change processes, including mindfulness, experiential avoidance, and valued living.

## 2. Materials and Methods

### 2.1. Participants

Participants were recruited from the VA Bedford Healthcare System, as well as via flyers and outreach to VA providers in the Boston metro area. A total of 143 U.S. veterans engaged in a brief phone screen for the present study. Of those veterans, 92 were invited to participate in an informed consent process and baseline intake to determine study eligibility. Inclusion criteria included the following: (1) current DSM-5 PTSD, (2) minimum score of 31 on the PTSD Checklist (PCL-5), (3) self-reported clinically significant difficulties in interpersonal relationships, (4) competent to provide written informed consent, (5) ages 18 and older. Exclusion criteria included any of the following: (1) any current or lifetime DSM-5 psychotic disorder, (2) current or recent (within 1 month of study entry) DSM-5 substance use disorder, (3) cognitive impairment that would interfere with study participation (e.g., dementia, intellectual disability, brain damage), (4) recent clinically significant suicidality (past 3 months), (5) moderate to severe domestic violence, and (6) participation in concurrent PTSD counseling.

**Figure 1:**
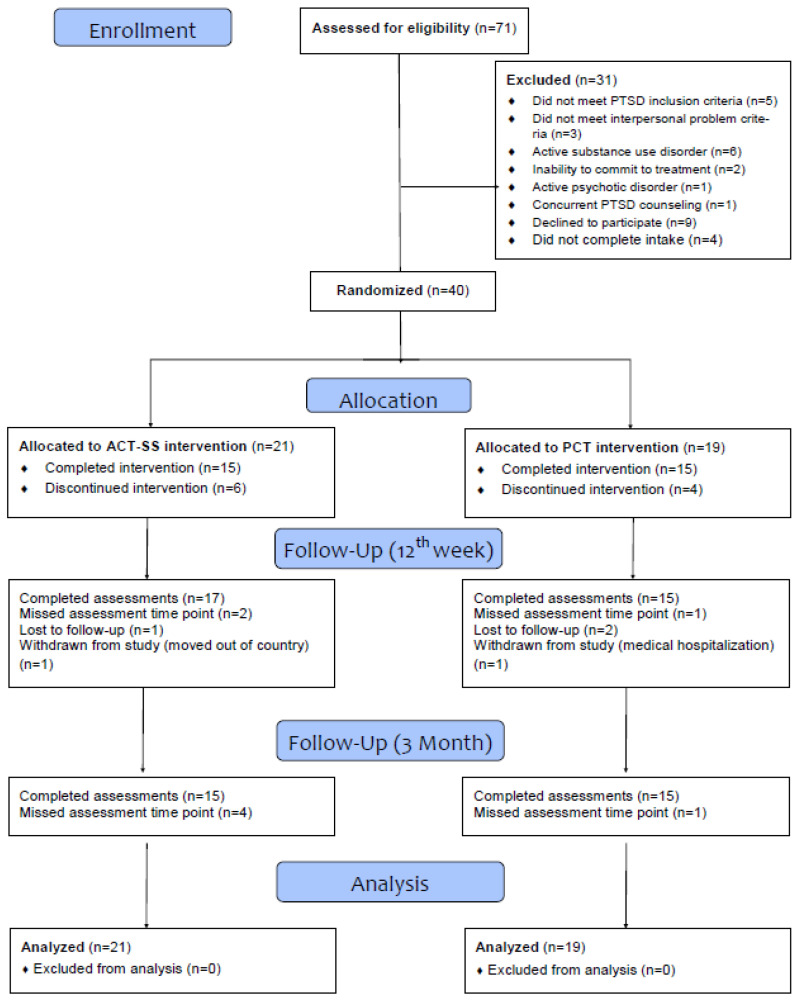
Consort diagram.

A total of 71 veterans at the VA Bedford Healthcare System completed an in-person evaluation to determine eligibility for the study. This study was conducted in accordance with the Declaration of Helsinki and approved by the VA Bedford Healthcare System IRB committee. There was a complete discussion of the study with potential participants and all participants signed statements of written informed consent after this discussion. A total of 31 participants were screened out at the baseline intake. Eighteen were screened out for the following reasons: did not meet criteria for PTSD (*n* = 5), did not report having clinically significant interpersonal problems (*n* = 3), active substance use disorder (*n* = 6), psychotic disorder (*n* = 1), concurrent PTSD counseling (*n* = 1), and inability to commit to treatment (*n* = 2). Nine other participants declined to participate in the study before randomization. Four other participants did not complete the intake. The final sample comprised 40 participants who began treatment. See Figure 1 for the CONSORT diagram of participant flow.

### 2.2. Measures

Measures were completed by both the ACT-SS and PCT groups at baseline, end of treatment (12th week), and at a three-month follow-up assessment.

A demographics assessment included questions about age, ethnicity, race, highest level educational attainment, yearly income, marital status, sexual orientation, and gender identity.

The Structured Clinical Interview for DSM-5 (SCID-5) was used for diagnosing mental health disorders for inclusion and exclusion criteria evaluation and to gather baseline mental health information to characterize the sample [42]. The SCID is a widely used structured interview that is used to diagnose mental health disorders with studies showing that the SCID yields highly reliable diagnosis for mental health disorders [43].

The Social Adjustment Scale-Self Report (SAS-SR) [44] is a 54-item measure of current social functioning in 6 domains: Work; Social and Leisure; Extended Family; Primary Relationship; Parental; and Family Unit. The SAS-SR is sensitive to change and has good convergent and discriminant validity. In the present study, the social and leisure subscale was used to evaluate change in the quantity and quality of social relationships and leisure activities not related to romantic relationships (nine items). Each question is rated using response scales specific to each of the nine items, with higher scores denoting greater impairment. Sample items for this subscale include, “How many friends have you seen or spoken to on the telephone in the last two weeks?”, “How many times in the last two weeks have you gone out socially with other people?”, and “Have you felt lonely and wished for more friends during the last two weeks?” Other subscales could not be reliably used given that a number of veterans did not have family relationships, current employment, a primary relationship, or parental relationships to report. The SAS-SR social and leisure subscale (Cronbach’s α = 0.71) demonstrated acceptable reliability in the present sample.

The Medical Outcomes Study Social Support Survey (MOS-SS) is a 19-item multidimensional, self-administered survey of social support for individuals with chronic conditions [45]. The MOS-SS measures four areas of social support: emotional/informational support, tangible support, positive social interaction, and affectionate support. Items assess how often raters are provided with companionship, assistance, or support, and are scored on a five-point Likert scale, from 1 (none of the time) to 5 (all of the time). An overall index score was calculated, with the mean item scores transformed to a 0–100 scale. The MOS-SS showed excellent internal reliability (Cronbach’s α = 0.97) for this sample.

The Quality of Life Enjoyment and Satisfaction Questionnaire Short Form (Q-LES-Q-SF) is a self-report measure commonly used to assess quality of life in several domains: general activities, physical health, subjective feelings, leisure time activities, social relationships, work, and household duties [46,47]. Items are rated on a 5-point Likert scale, from zero (very poor) to five (very good). Higher scores indicate better enjoyment and satisfaction with life. The scoring of the Q-LES-Q-SF involves summing the first 14 items to yield a total score, with total score ranging from 14 to 70 and expressed as a percentage based on the maximum total score of the items completed (0–100). In this study, we analyzed the overall Q-LES-Q score and the social relationships subscale to evaluate the change in the quality of social relationships.

The PTSD Checklist (PCL-5) [48,49,50] is a self-report symptom checklist that measures 20 symptoms of PTSD as defined in the DSM-5, and was used to assess PTSD for inclusion criteria as well as changes during and after treatment. The scale provides four subscale scores for PTSD symptom clusters, including: Cluster B (intrusion symptoms), Cluster C (avoidance of trauma-related stimuli), Cluster D (negative thoughts or feelings), and Cluster E (trauma-related arousal and reactivity) symptoms. Clients rate the degree to which they are bothered by each listed symptom of PTSD on a 5-point Likert-type scale, from 0 (not at all) to 4 (extremely). Items are summed to create a total score ranging from 0 to 80. Psychometric research suggests that a PCL-5 scores ≥ 31 in veterans indicates the presence of a PTSD diagnosis [51]. The PCL-5 has shown a satisfactory temporal stability, internal consistency, test–retest reliability, and convergent validity [48]. For the current study, the PCL-5 had good internal reliability (Cronbach’s α = 0.86).

The Mindful Attention Awareness Scale (MAAS) is a 15-item scale that measures the ability to mindfully observe the present moment [52]. Items assess how frequently raters report an open, receptive awareness of and attention to what is taking place in the present moment. Response options range from one (almost always) to six (almost never). Internal consistency levels (Cronbach’s alphas) generally range from 0.80 to 0.90. The MAAS has demonstrated high test–retest reliability, discriminant and convergent validity, known-groups validity, and criterion validity. For the present sample, the MAAS showed good internal reliability (Cronbach’s α = 0.83).

The Acceptance and Action Questionnaire-II (AAQ-II) [16] is a widely-used 7-item self-report measure of emotional avoidance and inaction. Items are ranked on a 7-point Likert scale, from 1 (never true) to 7 (always true), and items are summed to create a total score. The AAQ-II has good reliability and validity, with scores concurrently, longitudinally, and incrementally associated with and predictive of a range of outcomes, including mental health outcomes and work absence rates. In this study, the AAQ-II showed a good internal reliability (Cronbach’s α = 0.86).

The Valued Living Questionnaire (VLQ) is a two-part self-report measure of the extent to which a person is living in accordance with their values in everyday life [53,54]. The scale presents ten values domains: family, friend/social life, marriage/couples/intimate relations, parenting, work, education/training, recreation/fun, spirituality, citizenship/community life, and physical self-care. First, participants rate each domain according to how important the value is (importance), and then rate each domain on how consistently they have lived towards that value (consistency). A composite score is computed by multiplying the importance and consistency scores for each domain and averaging these products. Higher composite scores reflect taking actions that are more reflective of values.

The Client Satisfaction Questionnaire-8 (CSQ-8) is an 8-item client self-report scale that reflects a global satisfaction with and perceived quality of the effectiveness of mental health services [55]. It is scored on a 4-point Likert scale from one (poor) to four (excellent), with a total score range of 8 to 32. It has good reported internal consistency (Cronbach’s alpha = 0.83–0.93) [56] and showed good internal reliability within our sample (Cronbach’s α = 0.87).

The Working Alliance Inventory-Short Form (WAI-S) [57] is a 12-item scale administered at week 12 to obtain a working alliance assessment. The scale measures agreement between the patient and therapist across three main aspects of therapeutic alliance: the development of an affective bond, setting goals, and developing working tasks or activities. Each item is evaluated using a scale that ranges from one (never) to seven (always). It shows acceptable internal consistency (Cronbach’s α = 0.83–0.98) [57] and predictive validity related to therapy outcome [57,58,59]. The WAI-S (Cronbach’s α = 0.81) demonstrated good reliability in the present sample.

### 2.3. Procedures

*Screening Procedures*. Veterans were initially screened using a brief phone screen to obtain information relevant to the inclusion/exclusion criteria before study consent and intake. Veterans eligible after this screen were scheduled for an in-person assessment to obtain informed consent and confirm study eligibility. Subjects then completed self-report measures and participated in clinician-administered measures.

Veterans who met eligibility criteria consented and completed the intake process were randomized to ACT-SS or PCT. Veterans with either condition received twelve 50 min weekly sessions of individual treatment.

*ACT-SS Treatment.* The following components are emphasized in ACT-SS: *(1) Identifying Problems with Social Avoidance*: Participants identify efforts to avoid interpersonal experiences. Discussions focus on how avoidance is problematic for developing and maintaining relationships. *(2) Triggers for Avoidance*: Negative thoughts and emotions that lead to poor functioning and low quality of life are identified (e.g., worry over rejection, not being able to trust others, anger, feelings of being unworthy), and veterans practice acceptance and mindfulness to manage these experiences. *(3) Acceptance:* Veterans are encouraged to accept interpersonal situations that trigger concerns with mindful acceptance, rather than avoiding them. *(4) Mindfulness*: Participants engage in mindfulness exercises in order to practice nonjudgmental acceptance of their thoughts about others and negative emotions (e.g., leaves on a stream exercise, mindfulness of anger). *(5) Self-Compassion*: Veterans are encouraged to view themselves with more compassion, and practice self-compassion exercises (e.g., view themselves as a child who needs compassion). *(6)* *Valued Living*: Participants clarify their values and goals (i.e., building relationships, work achievement, community participation), and identify barriers that prevent them from achieving life goals. *(7) Willingness Exercises (Exposure)*: Participants develop hierarchies for interpersonal triggers and avoided social experiences and practice mindful acceptance during scheduled willingness exercises. *(8) Cognitive Defusion*: Participants learn that they are not their anxieties or fears, and they mindfully observe and accept these internal experiences. *(9) Committed Action*: Participants identify life goals and increase activities to improve social functioning, quality of life, and social reintegration. Participants commit to achieving valued goals.

To more directly meet the needs of veterans with PTSD and social relationship difficulties, specific modifications to ACT were made (see Table 1). *First*, PTSD is associated with a number of *interpersonal challenges*, including (1) rejection sensitivity [60]; (2) poor self-esteem [61]; and (3) being distrustful of others [62]. ACT-SS incorporates traditional ACT strategies and includes specific strategies to target poor social functioning. For example, adaptations include: (1) acceptance and mindfulness exercises about fears of being rejected by others, that others are threatening, feelings of being inadequate, and being distrustful of others; (2) identifying how social avoidance related to PTSD symptoms negatively affects social functioning, with willingness (exposure) exercises focused specifically on decreasing social avoidance and increasing community participation; (3) the incorporation of committed action exercises around increasing social interaction with others (a new socially focused goal every week). *Second*, we also included self-compassion exercises and a focus on forgiveness of the self and others to reduce the negative focus on low self-worth as a reason for avoiding others. *Third*, we included material on how to build healthy relationships, including specific skills for interacting with others (e.g., be present, validate the other person, be compassionate, share valued activities, and practice connection). *Fourth*, we included content on managing anger in an ACT-consistent manner, since anger is a key emotional barrier to developing and managing healthy relationships. Veterans practice being more mindful of anger and choose their actions based on their values and not on anger itself. *Finally*, we included content on trust in relationships, which is a major barrier to healthy interactions with others. A description of the ACT-SS treatment and a case study by Kelly and colleagues includes more details of this treatment approach [41].

Participants received 12 weekly 50 min individual counseling sessions. Session 1 was devoted to an explanation of the treatment rationale and identifying interpersonal triggers. Sessions 2–6 focused on mindfulness, cognitive defusion, and acceptance of PTSD symptoms, anxiety over interacting with others, and acceptance of other negative thoughts and emotions. Sessions 7–11 focused on self-compassion, relating to others, values, anger, and forgiveness, with a large emphasis on committed action and exposure hierarchies for social anxiety and avoidance. In the termination phase (sessions 11–12), therapy focused on termination, planning for the future, as well as reviewing progress and gains in treatment.

*Control Condition.* Person-Centered Therapy was the control condition, which is a commonly used treatment for discussing interpersonal difficulties. PCT is a non-directive approach that encourages participants to find their own solutions to their problems, including interpersonal problems. PCT is focused on creating a safe, non-judgmental therapeutic environment in which therapists engage in active listening and demonstrate unconditional positive regard for their clients. Participants are encouraged to explore their own experiences and emotions in order to make their own decisions about how they need to change. PCT consisted of the same dosage and intensity of psychosocial treatment as ACT-SS. PCT was delivered in 12 50-min weekly sessions, matched to the length and frequency of ACT-SS sessions.

*Therapists and Treatment Integrity.* ACT-SS and PCT was provided by psychologists or postdoctoral residents. The first author provided training in both ACT-SS and PCT via workshops for each type of psychotherapy. Study therapists had weekly supervision with Dr. Kelly to check protocol adherence. Each case was reviewed weekly. At least four randomly selected sessions with each therapist were discussed in detail with the first author to assess treatment adherence. There were no protocol violations. Therapist competence was not rated.

*Statistical analyses.* We assessed the feasibility and acceptability of ACT-SS for veterans with PTSD vs. PCT as a control condition by examining rates of treatment completion. We also examined reasons for withdrawing from treatment for consistent patterns. Overall treatment satisfaction was measured with the CSQ-8-R and the quality of the therapeutic alliance on the WAI-S. We also collected qualitative data on the acceptability of ACT-SS using structured individual interviews at the end of treatment, which asked about exercises that were the most helpful, whether they would recommend the treatment to others, and perceived changes as a result of treatment.

Given that this was a pilot study, there is not enough power to test hypotheses regarding the potential between-condition differences in outcome measures. In addition, we explored differences in both treatment groups over time using a repeated measures ANOVA on the quality of social relationships (Q-LES-Q), levels of social support (MOS-SS), engagement in social and leisure activities (SAS-SR), and PTSD symptoms (PCL-5). We also examined the effects of treatment on potential mediators (i.e., process variables—MAAS, AAQ, VLQ), and the effect of these mediators on treatment outcomes to identify candidate mediators for future study. Based on the intent-to-treat principle, all participants randomized to treatment were included in the analyses.

## 3. Results

### 3.1. Demographic Characteristics

The demographic characteristics of the sample are presented in Table 2. The sample had an average age of 55.8 years (*SD* = 11.9) and were primarily Caucasian (70.0%), followed by Black/African American (15.0%) and American Indian/Alaska Native (5.0%). The sample was also primarily non-Hispanic (77.5%). The majority of participants identified as male (87.5%) and straight/heterosexual (92.5%) The majority of participants were never married, widowed, separated, or divorced (60%) and had at least some college education or higher education (75%). The mean annual income of the sample was low (*M* = USD 9682, *SD* = USD 20,344). All participants had a lifetime history of a depressive disorder, followed by high lifetime rates of anxiety disorders (77.5%) and substance use disorders (50.0%). There were no differences between the ACT-SS and PCT groups for any of the demographic characteristics.

### 3.2. Treatment Retention

There were no differences in treatment completion between the ACT-SS and PCT groups. In the ACT-SS condition, 71% of veterans completed all 12 sessions of treatment (*n* = 15), and 29% discontinued treatment (*n* = 6). In the PCT condition, 79% of veterans completed all 12 sessions of treatment (*n* = 15) and 21% discontinued treatment (*n* = 4). In the ACT-SS condition, of the six veterans who did not complete treatment, one had increased job responsibilities and could no longer participate in the treatment, one lost transportation, one had a family emergency to attend to, and two did not respond to calls or a letter. In the PCT condition, of the four participants who discontinued treatment, one moved away, one had a medical hospitalization that interfered with treatment, and two did not respond to calls or a letter. There did not appear to be a consistent pattern of reasons for drop out in either the ACT or PCT conditions.

### 3.3. Feasibility and Acceptability of Treatment

Client satisfaction was rated using the CSQ-8 for treatment completers. There were no differences between the ACT-SS (*M* = 29.1, *SD* = 2.56) and PCT groups (*M* = 29.9, *SD* = 4.04) on the CSQ-8. Overall, client satisfaction for both treatments was high. There were no differences between the ACT-SS group (*M* = 74.42, *SD* = 4.19) and PCT group (*M* = 72.64, *SD* = 11.37) in the Working Alliance Inventory for those who completed treatment.

When asked about what perceived changes they noted about themselves, ACT-SS participants reported that they were less critical of themselves and others, more open and willing to do things that were uncomfortable, and more active in social activities. Please see Table 3 for representative quotes from participants. Qualitative feedback indicated that veterans would recommend the ACT-SS treatment to others. Representative quotes included: “I would recommend it because it helped me to grow and I think it could help someone else”, “Absolutely, because it’s useful. I think even someone without PTSD could really benefit from this. Especially men in a military mindset”, “Yes, because it gets them to open their mouth, develop a sense of trust, and creates a discipline with how to deal with fears, doubts and insecurities”, and “Yes, it does two things, (1) talking to somebody other than your friends and family, (2) it’s directed towards achieving certain goals, not just talk therapy.”

Of the eleven veterans who answered questions about which ACT exercises were the most helpful, the most popular exercise (45%) was “Joe the Problem”, which frames difficult internal experiences as an unwelcome party guest to be accepted in the service of staying engaged at the party (valued life activities). Other popular exercises included the finger trap, which illustrates the paradoxical effect of trying to suppress thoughts and emotions versus willingly experiencing them (36%); passengers on a bus, which presents difficult thoughts and emotions as passengers who, if given control, will divert the driver from valued life directions (36%); and sky/weather, which teaches participants to view themselves as the ever-present context (i.e., the sky) for thoughts and emotions (i.e., the weather; 27%). There was no consistent pattern of any ACT exercises being the least helpful. Of the same group of veterans, 82% of veterans reported that mindfulness exercises were helpful, with 45% indicating that the leaves on the stream meditation and mindfulness of the breath meditation were particularly helpful. A representative quote about mindfulness exercises was, “All of them were helpful. They are key to when you decide to let go and try to relax and get clarity about what is really going on. It has always been helpful. It allows me to let go of a lot of things affecting me”.

### 3.4. Treatment Outcomes

Overall, there were no differences in the Q-LES-Q summary score at the end of treatment (ACT-SS *η^2^_p_* = 0.155, PCT *η^2^_p_* = 0.036) or the three-month follow-up (ACT-SS *η^2^_p_* = 0.187, PCT *η^2^_p_* = 0.032) (see Table 4). However, for the ACT-SS group, the quality of social relationships significantly improved from baseline to the end of treatment [*F*(1, 20) = 7.56, *p* = 0.012, *η^2^_p_* = 0.274)] and the three-month follow-up [*F*(1, 20) = 7.16, *p* = 0.015, *η^2^_p_* = 0.264)]. No differences at the end of treatment (*η^2^_p_* = 0.042) or three-month follow-up (*η^2^_p_* = 0.000) were observed for the PCT group on the quality of social relationships on the Q-LES-Q.

There were no differences from baseline to the end of treatment (ACT-SS *η^2^_p_* = 0.001, PCT *η^2^_p_* = 0.041) or the three-month follow-up (ACT-SS *η^2^_p_* = 0.000, PCT *η^2^_p_* = 0.024) in either condition on the MOS Social Support Survey. For the ACT-SS group, there was an improvement in engagement in social and leisure activities from baseline to the end of treatment [*F*(1, 20) = 4.49, *p* = 0.047, *η^2^_p_* = 0.183] and from baseline to the three-month follow-up, [*F*(1, 20) = 5.46, *p* = 0.030, *η^2^_p_* = 0.215]. There were no differences for engagement in social and leisure activities for the PCT group from baseline to either the end of treatment (*η^2^_p_* = 0.073) or the three-month follow-up (*η^2^_p_* = 0.001).

In the ACT-SS group, PTSD symptoms of the PCL-5 significantly decreased from baseline to the end of treatment [*F*(1, 20) = 6.91, *p* = 0.016, *η^2^_p_* = 0.257] and continued to remain significantly decreased the three-month follow-up [*F*(1, 20) = 9.59, *p* = 0.006, *η^2^_p_* = 0.324]. However, for the PCT group, PTSD symptoms did not differ between baseline and either the end of treatment (*η^2^_p_* = 0.099) or the three-month follow-up (*η^2^_p_* = 0.051).

In the ACT-SS group, there was a significant improvement in mindfulness, attention, and awareness on the MAAS from baseline to the end of treatment [*F*(1, 20) = 4.61, *p* = 0.044, *η^2^_p_* = 0.187], but not from baseline to the three-month follow-up (*η^2^_p_* = 0.075). There were no differences in mindfulness, attention, and awareness in the PCT group from baseline to either the end of treatment (*η^2^_p_* = 0.063) or three-month follow-up (*η^2^_p_* = 0.010). Similarly, there was a significant reduction in psychological inflexibility as measured by the AAQ-II from baseline to the end of treatment for the ACT-SS group [*F*(1, 20) = 4.73, *p* = 0.042, *η^2^_p_* = 0.191], but no difference between baseline the three-month follow-up for the ACT-SS group (*η^2^_p_* = 0.093), or for either the end of treatment (*η^2^_p_* = 0.002) or three-month follow-up (*η^2^_p_* = 0.004) for the PCT group. Finally, there was a significant improvement in values-based living on the VLQ for the ACT-SS group for both the baseline to the end of treatment [*F*(1, 20) = 5.83, *p* = 0.025, *η^2^_p_* = 0.226] and the three-month follow-up [*F*(1, 20) = 4.68, *p* = 0.043, *η^2^_p_* = 0.190]. However, no differences in values-based living were observed for the PCT group at either the end of treatment (*η^2^_p_* = 0.016) or the three-month follow-up (*η^2^_p_* = 0.117).

## 4. Discussion

This pilot randomized controlled trial provides important evidence for the feasibility and acceptability of an ACT intervention designed to improve the social relationships of veterans with PTSD. Study metrics including therapy retention and satisfaction, as well as qualitative feedback from participants, demonstrated the feasibility and acceptability of the intervention. Secondary analyses also provided preliminary support for the effects of the intervention on key outcomes and potential change processes for the intervention. Together, these findings demonstrate the promise of ACT-SS for targeting low levels of social support among veterans with PTSD, a population at high risk of mortality and other negative outcomes [1,2,3,4].

Overall, participants in both conditions reported satisfaction with treatment, suggesting the non-inferiority of ACT-SS compared to the active control intervention for this index of acceptability. Furthermore, there was less than a 30% dropout in both conditions, which is particularly meaningful for a sample of veterans with PTSD who demonstrated marked social avoidance and reported interpersonal difficulties and social isolation. In qualitative interviews, participants in the ACT condition indicated that they would recommend the treatment to others. In particular, participants reported that they thought that mindfulness exercises, such as mindful breathing and leaves on a stream were helpful to create awareness of their thoughts and emotions when interacting with others. Participants also indicated that the intervention helped them feel more open and willing to interact with others, even if it was difficult. They reported increased insight into their own thoughts, emotions, and actions, which helped them avoid automatically acting on their thoughts and emotions, leading to improved relationships.

The preliminary results of this study showed that although ACT-SS did not lead to increased overall quality of life, it was associated with an increased quality of social relationships, likely because this is a direct target of this intervention. The increased quality of social relationships in the ACT-SS group were also extended to the three-month follow-up, demonstrating at least a short-term sustained improvement. The ACT-SS intervention focused on the development of a long-term game plan for improving social relationships, which may have helped veterans to sustain their social relationships after treatment. In addition, this result was supported by an increased engagement in social and leisure activities at both the end of treatment and three-month follow-up for the ACT-SS group. No changes over time in either of these variables were observed in the PCT condition, which does not explicitly target social relationships in treatment.

There were no changes observed in either the ACT-SS group or PCT group for the MOS Social Support Survey. One major reason for this result may be that the MOS Social Support Survey [45] asks the respondent to identify “someone” who can provide advice, understands problems, can provide assistance, and with whom they can practise enjoyable activities. Many veterans in this study had at least one person in their lives that they could enjoy things with and that they could count on. However, they still reported feeling socially isolated from their community and wanting to increase their social activities and the quality of their overall relationships. As a result, the MOS Social Support Survey may not have been sensitive enough to assess global social support needs, rather asking people to identify only one person that provides support. However, given that ACT-SS conceptually has more of an impact on social connectedness rather than MOS-defined social support, it may be worth considering renaming the treatment to be more aligned with the actual target of treatment.

The results of this study support the research on ACT interventions demonstrating improvement in social relationships. For instance, a study of a 10-session ACT intervention targeting the interpersonal problems of university students in Iran (*n* = 66) showed a significant reduction in interpersonal problems and emotion regulation difficulties [35]. A pilot study of a 12-session ACT treatment for mental health carers (*n* = 24) showed that ACT resulted in significant reductions in interpersonal problems and caregiving avoidance, and increased mindfulness, psychological flexibility, and wellbeing over time [36]. Qualitative results from this study also showed that participants reported improvements in reactivity, communication, agency, and connection to others. Another study that examined the effect of a 10-session ACT group intervention for high school students with social anxiety disorder (*n* = 30) [34] showed significant improvements in interpersonal problems and a reduction in psychological inflexibility, consistent with the results of the present study. The aforementioned studies and the present study mostly had small sample sizes, which limits the ability to draw firm conclusions about the effect of ACT on relationships. Although the results from this pilot study and other ACT interventions on interpersonal problems are promising, larger randomized controlled trials are necessary to more adequately test the efficacy of ACT interventions that target interpersonal problems and improvements in social relationships.

Furthermore, results from this study showed that there was a significant reduction in PTSD symptoms at both the end of treatment and the three-month follow-up for the ACT-SS group, but not in the PCT group. It is notable that PTSD symptoms were not directly targeted in this study, and no traditional exposure exercises for PTSD symptoms were utilized (e.g., repeated recounts of the trauma, as in prolonged exposure), but veterans in the ACT-SS group still experienced a statistically and clinically significant symptom reduction of about 10 points in the PCL-5. The emphasis on engaging in avoided social activities may have provided veterans with important exposure to previously avoided situations akin to in vivo experiences of prolonged exposure. This focus on previously avoided situations that were also connected with veterans’ values and the desire for increased connection may have provided an opportunity for participants to experience a reduction in PTSD symptoms.

Although there are case studies that have shown the promise of ACT for people with PTSD [37,38], few empirical trials demonstrated a significant improvement in PTSD symptoms as a result of an ACT intervention. A pilot study of 12-session ACT group and individual interventions showed a significant improvement in PTSD symptoms in both the ACT group (*n* = 10) and individual interventions (*n* = 9) [39]. Another pilot study of a web-based ACT intervention in trauma-related psychological difficulties for interpersonal trauma survivors (*n* = 25) showed a significant reduction in PTSD symptoms [63]. An open trial of a 12-session ACT intervention for co-occurring PTSD and alcohol use disorder (*n* = 43) showed an improvement in clinician-assessed and self-reported PTSD symptoms and quality of life post-treatment and at a 3-month follow-up [40]. However, as mentioned above with regard to ACT interventions for interpersonal difficulties, more research with larger randomized controlled trials is necessary to better understand the effects of ACT on individuals with PTSD.

All three process variables showed a significant change in the ACT-SS group from baseline to the end of treatment, including an improvement in mindfulness and valued living and a reduction in experiential avoidance. These changes were not evident in the PCT (active control) group, suggesting that ACT-SS effectively targeted the hypothesized change processes and provided support for these candidate mediators in future, larger-scale trials of the study intervention. The sustained improvement in valued living at the three-month follow-up is consistent with research suggesting that values work is an important component of ACT interventions, and valued living is a key mediator of outcomes, especially those that are not specific to diagnostic symptoms. For example, changes in valued behaviors precede and influence changes in the subsequent levels of suffering [64], and dismantling research suggests that the values and committed action components of ACT are especially impactful for broad indicators of functioning [65], at times performing, along with full-model ACT interventions, for outcomes that include a social well-being component [66]. However, no studies evaluated how an emphasis on valued living as a process could specifically improve social functioning and relationships. Future studies on ACT for social relationships should evaluate these associations.

The present study was a small, randomized pilot study, which limits conclusions about treatment outcomes as the study was not sufficiently powered to detect specific treatment effects in comparison to control conditions. Future larger randomized controlled trials with blinded assessments are necessary to evaluate the efficacy of ACT-SS. In addition, although a careful weekly review of session details and the approach occurred for therapists throughout the study in both the ACT-SS and PCT conditions, therapist competence was not assessed using adherence measures. Future studies of ACT-SS should ensure that therapist adherence is systemically evaluated. In addition, we included only subjective measures of social engagement, and future studies should consider including more objective measures (e.g., analysis of the number of social activities). We also limited the treatment to 12 sessions, and it is unclear whether a longer duration of treatment would result in better outcomes. Finally, the sample was largely composed of non-Hispanic, white, heterosexual men, and future studies should evaluate the effects of ACT-SS on a broader range of veterans.

Although preliminary, these results suggest that the targeting of improved social relationships with ACT is a promising treatment approach for veterans with PTSD, acceptable to veterans, and feasible to use and evaluate in a research context. Further research is needed in order to determine the efficacy of ACT-SS for veterans with PTSD in larger randomized controlled trials.

## Figures and Tables

**Table 1:** ACT-SS Manual Content.

**Major Content Areas of ACT-SS**	
Identifying Problems with Social Avoidance	Veterans are asked to identify efforts to avoid interpersonal experiences and how this is problematic for developing and maintaining relationships
Identifying Triggers	Interpersonal triggers are identified, with an emphasis on how PTSD-related triggers and symptoms are related to interpersonal conflict and avoidance.
Acceptance and Mindfulness	Veterans participate in mindfulness exercises in order to practice nonjudgmental acceptance of interpersonal experiences and PTSD symptoms that interfere with interpersonal interactions (e.g., irritability and feelings of detachment).
Valued Living	Veterans clarify their values and goals (i.e., reasons for changing patterns in interpersonal experiences), and identify barriers (thoughts, feelings, sensations associated with PTSD) that prevent them from achieving these goals.
Cognitive Defusion	Veterans learn that partners, family members, and peers are not threats to their safety and to mindfully observe anxieties about social interactions.
Willingness Exercises	Veterans create exposure hierarchies for social interactions and face them with mindful acceptance to become more comfortable with these experiences.
Committed Action	Veterans incorporate more social activities in their lives and opportunities to interact with others that are consistent with valued goals. Veterans commit to spending more time with important social supports and develop new relationships.
**Specific Content Areas to Address for Veterans with PTSD in ACT-SS**	
Social Isolation	Goals focus on being willing to engage in social interactions and increasing community involvement, while accepting PTSD-related symptoms.
Building Healthy Relationships	Ask veterans to be present, validate the other person, be compassionate, share valued activities, and practice connection.
Anger	Veterans learn to be more mindful of anger and choose to act according to their values.
Trust	Veterans balance values around trust with values around self-protection and practice mindful trusting—be aware of the person’s behavior and provide trust when it is earned.

**Table 2:** Demographic characteristics at baseline (*n* = 40).

Characteristic	Total (*n* = 40)	ACT (*n* = 21)	PCT (*n* = 19)	*t* or χ^2^	*p*
Age	55.8 (11.9)	54.9 (10.7)	56.8 (13.3)	−0.501	0.619
Gender					
Female	12.5% (5)	19.0% (4)	5.3% (1)		
Male	87.5% (35)	81.0% (17)	94.7% (18)		
Other	0% (0)	0% (0)	0% (0)		
Sexual Orientation				0.261	0.609
Straight/Heterosexual	92.5% (37)	90.5% (19)	94.7% (18)		
Not Straight/Heterosexual	7.5% (3)	9.5% (2)	0% (0)		
Declined to Answer	2.5% (1)	0% (0)	5.3% (1)		
Race				0.043	0.836
White	70.0% (28)	71.4% (15)	68.4% (13)		
Black/African American	15.0% (6)	19.0 (4)	10.5% (2)		
American Indian/Alaska Native	5.0% (2)	4.8% (1)	5.3% (1)		
Asian American	0% (0)	0% (0)	0% (0)		
Native Hawaiian or other Pacific Islander	0% (0)	0% (0)	0% (0)		
Other	2.5% (1)	0% (0)	5.3% (1)		
Declined to Answer	10.0% (4)	9.5% (2)	10.5% (2)		
Ethnicity				0.478	0.489
Hispanic	7.5% (3)	4.8% (1)	10.5% (2)		
Not Hispanic	77.5% (31)	81.0% (17)	73.7% (14)		
Declined to Answer	15.0% (6)	14.3% (3)	15.8% (3)		
Highest Level of Education				0.416	0.519
Some High School or Less	5.0% (2)	0% (0)	10.5% (2)		
High School/GED	17.5% (7)	19.0% (4)	15.8% (3)		
Some College	22.5% (9)	14.3% (3)	31.6% (6)		
Associate’s/Vocational/Technical Degree	20.0% (8)	19.0% (4)	21.1% (4)		
Bachelor’s Degree	10.0% (4)	19.0% (4)	0% (0)		
Some Graduate School	7.5% (3)	4.8% (1)	10.5% (2)		
Graduate Degree	15.0% (6)	23.8% (5)	5.3% (1)		
Declined to Answer	2.5% (1)	0%(0)	5.3% (1)		
Mean Income	9682 (20,344)	9470 (23,071)	9933 (17,694)	−0.054	0.957
Marital Status				2.41	0.121
Never Married	12.5% (5)	19.0% (4)	5.3% (1)		
Married	40.0% (16)	28.6% (6)	52.6% (10)		
Widowed	2.5% (1)	0% (0)	5.3% (1)		
Separated	2.5% (1)	0% (0)	5.3% (1)		
Divorced	45.0% (18)	52.3% (11)	31.6% (6)		
Lifetime Psychiatric Disorders					
Bipolar Disorder	5.0% (2)	4.8% (1)	5.3% (1)	0.013	0.911
Depressive Disorder	100% (40)	100% (21)	100% (19)	---	---
Substance Use Disorder	50.0% (20)	52.4% (11)	47.4% (9)	0.227	0.634
Anxiety Disorder	77.5% (31)	76.2% (16)	78.9% (15)	0.007	0.935
Obsessive Compulsive and Related Disorders	10.0% (4)	9.5% (2)	10.5% (2)	0.027	0.871

Note. ACT-SS = Acceptance and Commitment Therapy to Improve Social Support for Veterans with PTSD; PCT = Person-Centered Therapy.

**Table 3:** Qualitative feedback from participants about perceived changes in themselves as a result of engaging in ACT-SS.

I feel like I am more open, more receptive, and more understanding of others’ needs.
I have better quality of life, I am doing more, leaving my house, and seeing people.
I am not beating myself up.
Positive changes. Willingness to try new things. Willingness to get out more.
I am more willing to talk with family about things, even if it’s uncomfortable, and I am willing to listen more. I am more willing to step out of my comfort zone and do more social activities, reaching out to friends more and not giving up right away.
I feel better about others and about myself.
Increasing insight/self-awareness, stop trying with people who do not care or who do not put in as much effort as I do.
I am allowing more self-trust. Allowing my feelings to come out. Life is not perfect. I am not perfect. Day to day recognition of where I am at and where I want to be. Letting go of negative thoughts. Not allowing anger to surface to the point that it is destructive.
Recognizing more clearly how I got to this. What I resist will persist. Identifying an internal dialogue on why I do certain things. Stepping outside of myself and having to do things—doing things I do not want to do because I have to for myself and others.

**Table 4:** Means and standard deviations of study outcome and process variables.

ACT-SS (*n* = 21)			PCT (*n* = 19)	
	*M*	*SD*	*M*	*SD*
Q-LES-Q-SF Total Score				
Baseline	41.7	9.2	40.7	8.7
End of Treatment	44.0	10.0	39.1	11.1
Three Month Follow-up	44.1	8.7	39.2	11.9
Q-LES-Q-SF Social Relationships				
Baseline	2.00	1.10	2.47	1.02
End of Treatment	2.52 *	1.21	2.26	1.10
Three Month Follow-up	2.57 *	1.12	2.47	1.07
MOS Social Support				
Baseline	45.2	25.6	59.8	25.5
End of Treatment	45.8	23.9	59.8	29.6
Three Month Follow-up	49.4	27.4	62.2	27.0
SAS-SR Social and Leisure Activities				
Baseline	3.29	0.71	3.11	0.98
End of Treatment	2.90 *	0.75	2.89	0.82
Three Month Follow-up	2.89 *	0.74	3.13	0.92
PTSD Symptom Checklist				
Baseline	49.7	12.7	46.3	9.5
End of Treatment	40.9 *	21.4	42.9	14.4
Three Month Follow-up	40.7 *	18.2	43.5	16.2
Mindfulness, Attention, and Awareness Scale				
Baseline	3.01	0.86	3.50	0.80
End of Treatment	3.41 *	0.94	3.31	1.10
Three Month Follow-up	3.24	0.79	3.40	1.17
Acceptance and Action Questionnaire				
Baseline	33.0	8.38	33.8	7.93
End of Treatment	28.9 *	8.91	34.1	8.99
Three Month Follow-up	29.9	8.74	34.3	9.29
Valued Living Questionnaire				
Baseline	37.3	19.9	37.8	17.3
End of Treatment	45.7 *	21.7	35.5	20.5
Three Month Follow-up	44.6 *	22.3	32.1	18.0

Note. * *p* < 0.05. Q-LES-Q-SF = The Quality of Life Enjoyment and Satisfaction Questionnaire Short Form; MOS Social Support = The Medical Outcomes Study Social Support Survey; SAS-SR = Social Adjustment Scale-Self Report.

## Data Availability

Data are available from the corresponding author upon request.
